# Global analysis of estrogen receptor beta binding to breast cancer cell genome reveals an extensive interplay with estrogen receptor alpha for target gene regulation

**DOI:** 10.1186/1471-2164-12-36

**Published:** 2011-01-14

**Authors:** Oli MV Grober, Margherita Mutarelli, Giorgio Giurato, Maria Ravo, Luigi Cicatiello, Maria Rosaria De Filippo, Lorenzo Ferraro, Giovanni Nassa, Maria Francesca Papa, Ornella Paris, Roberta Tarallo, Shujun Luo, Gary P Schroth, Vladimir Benes, Alessandro Weisz

**Affiliations:** 1 Department of General Pathology, Second University of Naples, vico L. De Crecchio 7, 80138 Napoli, Italy; 2 Molecular Medicine Laboratory, University of Salerno, via Allende, 84081 Baronissi, Italy; 3 Illumina, Inc., Hayward, 94545 California, USA; 4 Genomics Core Facility, European Molecular Biology Laboratory, Heidelberg 69117, Germany

## Abstract

**Background:**

Estrogen receptors alpha (ERα) and beta (ERβ) are transcription factors (TFs) that mediate estrogen signaling and define the hormone-responsive phenotype of breast cancer (BC). The two receptors can be found co-expressed and play specific, often opposite, roles, with ERβ being able to modulate the effects of ERα on gene transcription and cell proliferation. ERβ is frequently lost in BC, where its presence generally correlates with a better prognosis of the disease. The identification of the genomic targets of ERβ in hormone-responsive BC cells is thus a critical step to elucidate the roles of this receptor in estrogen signaling and tumor cell biology.

**Results:**

Expression of full-length ERβ in hormone-responsive, ERα-positive MCF-7 cells resulted in a marked reduction in cell proliferation in response to estrogen and marked effects on the cell transcriptome. By ChIP-Seq we identified 9702 ERβ and 6024 ERα binding sites in estrogen-stimulated cells, comprising sites occupied by either ERβ, ERα or both ER subtypes. A search for TF binding matrices revealed that the majority of the binding sites identified comprise one or more Estrogen Response Element and the remaining show binding matrixes for other TFs known to mediate ER interaction with chromatin by tethering, including AP2, E2F and SP1. Of 921 genes differentially regulated by estrogen in ERβ+ *vs *ERβ- cells, 424 showed one or more ERβ site within 10 kb. These putative primary ERβ target genes control cell proliferation, death, differentiation, motility and adhesion, signal transduction and transcription, key cellular processes that might explain the biological and clinical phenotype of tumors expressing this ER subtype. ERβ binding in close proximity of several miRNA genes and in the mitochondrial genome, suggests the possible involvement of this receptor in small non-coding RNA biogenesis and mitochondrial genome functions.

**Conclusions:**

Results indicate that the vast majority of the genomic targets of ERβ can bind also ERα, suggesting that the overall action of ERβ on the genome of hormone-responsive BC cells depends mainly on the relative concentration of both ERs in the cell.

## Background

Estrogens are key regulators of cell growth and differentiation in the mammary gland [1,2], where they are involved in the pathogenesis and clinical outcome of breast cancer (BC) [3]. These steroid hormones exert their effects in normal and transformed mammary epithelial cells by binding to specific receptors, ERα and ERβ, that mediate estrogen signaling by functioning as ligand-dependent transcription factors. Ligand-activated ERs drive gene cascades comprising primary genes, whose transcription is directly controlled by the hormone through physical interaction of ERs with regulatory sites in the genome (genomic pathway) and/or with signal transduction effectors (non genomic pathway), as well as downstream genes whose activity depends upon the functions encoded by the primary responders [1,4].

ERs are able to bind DNA at specific sites in the genome and thereby control gene activity by recruiting transcriptional mediators and co-regulators, as well as a host of other nuclear proteins with different roles in ER-mediated control of gene activity [5,6]. The two ERs show 55% identity in their estrogen-binding domains (LBDs) and approximately 97% similarity in the DNA-binding domains (DBDs) [7]. Reflecting the high degree of similarity in their DBDs, both receptors interact with the same conserved estrogen response element (ERE) (5'-GGTCAnnnTGACC-3') as either homodimers or alpha/beta heterodimers [8,9]. ERβ, however, holds low trans-acting capability on ERE-containing estrogen target genes and alpha/beta heterodimers are less efficient than ERα homodimers in promoting target genes activity [10]. The different behaviour of ERα/ERβ heterodimers respect to ERα homodimers on transcriptional regulation of ERE-containing genes might be explained by different co-factor recruitment, as ERβ could prevent efficient co-activator binding to the ERα moiety of the heterodimer, conversely inducing recruitment of co-repressors and/or driving assembly of co-regulatory complexes other than those involving ERα only. [8,11,12].

Although the two receptors are quite similar in sequence and structure, in BC ERβ has considerably different biological effects than ERα [1,13,14]. Furthermore, the two ERs show a remarkably different expression pattern in BCs, with higher ERα and lower ERβ levels observed in malignant cells compared to normal mammary epithelial or benign tumor cells [15,16]. Furthermore, while ERα induces a mitogenic response to estrogen, when expressed alone the β subtype is not only unable to induce the same mitogenic response, but it reduces basal, hormone-independent cell proliferation [17-18 and R. Tarallo *et al.*, unpublished]. Finally, ERβ was shown to change dramatically ERα-positive BC cell behaviour *in vivo*, as its expression in the cell prevents tumorigenicity in mouse xenograft models by reducing tumor growth and angiogenesis [19,20].

Gene expression studies performed in BC cell lines expressing endogenous ERα and recombinant ERβ [21-23] revealed multiple signaling pathways involving the α and/or β receptor subtypes [1]. The two ERs appear thus to share many target genes, although each of them may affect specific downstream targets. For this reason, inhibition of hormone-responsive BC cell growth by ERβ might be due to direct interference with ERα activity on growth-promoting pathways as well as to the activity of ERβ-specific target genes [24].

Recently, next-generation sequencing technologies combining chromatin immunoprecipitation (ChIP) either with genomic DNA hybridization to microarrays (ChIP-on-chip) or massively parallel sequencing (ChIP-Seq, ChIP-PET), opened new venues for our understanding of physical and functional associations between transcription factors and chromatin *in vivo*. These analytical strategies led to genome-wide mapping of ERα-binding regions in intact chromatin of cultured cell lines [25-28], revealing important new information relative to ERα interaction with the genome. Carroll *et al. *[25], for example, using ChIP-on-chip demonstrated that the Forkhead factor FoxA1 plays an important role as pioneering factor for ERα binding to chromatin in BC cells, while Cicatiello *et al. *[26] identified novel gene regulation cascades mediating estrogen actions in hormone-responsive BC cells. In contrast, although several studies focused on ERβ interaction with the genome [29-32], a thorough characterization of this important aspect of ERβ biology in BC cells, essential to clarify the mechanisms mediating its control of estrogen-dependent gene pathways and the hormone-responsive phenotype, is still missing. For this reason, we performed a comprehensive analysis of ERβ and ERα target sites in the genome of MCF-7 cells engineered to express both receptors to comparable levels, by integrating global mapping of *in vivo *ER binding to the genome by ChIP-Seq with comparative gene expression profiling in ERβ+/ERα+ *vs *ERβ-/ERα+ cells during early stimulation with 17-beta-estradiol (E2), followed by *in silico *analyses of the ERβ binding regions and responsive genes identified.

## Results and Discussion

### Establishment and characterization of ERβ-expressing MCF-7 cells

**Figure 1:**
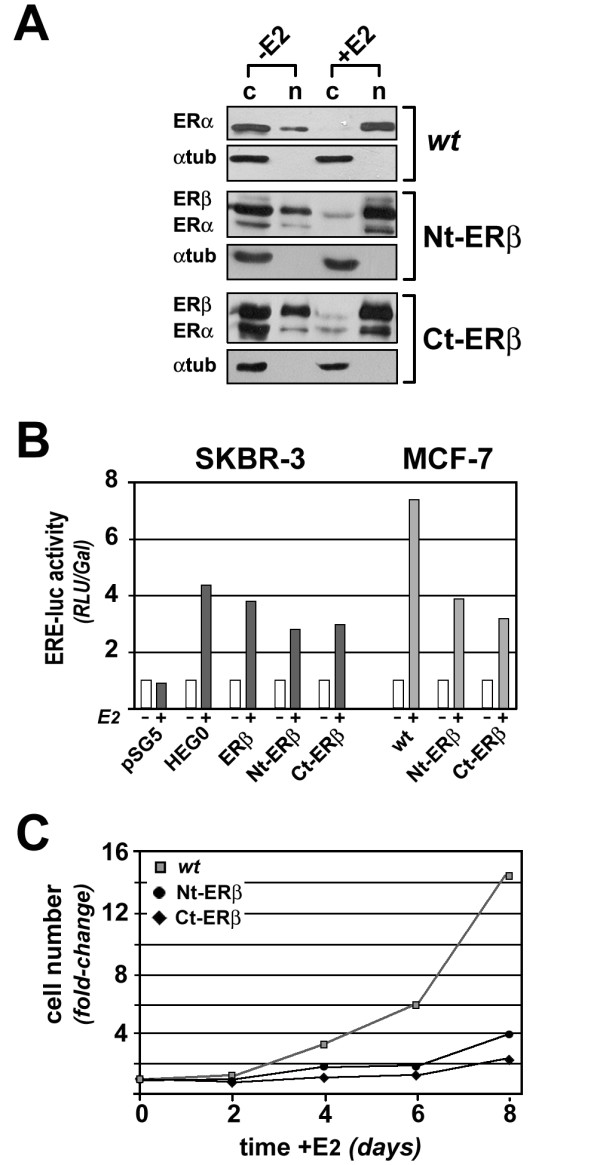
**Functional characterization of ERβ-expressing MCF-7 cells**. (A) Nuclear translocation of ERα and ERβ shown by western blot analysis on cytosolic (c) and nuclear (n) protein extracts, prepared from *wt *MCF-7, Nt-ERβ and Ct-ERβ cells after treatment with either 17β-estradiol (10^-8^M, +E2) or vehicle alone (-E2) for 45 minutes. The amount of α-tubulin was also analyzed to verify the absence of cytosolic contaminants in the nuclear fractions. (B) The transcriptional activity of ERα, Nt-ERβ and Ct-ERβ was measured by transient transfection in SKBR3 cells (*left*) and the ability of tagged ERβ to interfere with ERα activity was assessed by comparing estrogen effects in wt, in Nt-ERβ and Ct-ERβ MCF-7 cells (*right*); in all cases transiently transfected ERE-*tk*-luc was used as reporter gene. (C) Proliferation of *wt *MCF-7, Nt-ERβ and Ct-ERβ cells was measured by stimulating hormone-starved cells with 10^-8^M E2, followed by cell counting with a colorimetric assay at the indicated times.

Stabilized human BC cell lines expressing endogenous ERα and ERβ at comparable levels are not available. For this reason, we first generated and characterized cell lines derived from ERα-positive MCF-7 cells expressing full-length human ERβ (ERβ1) at levels similar to those of endogenous ERα. This strategy was adopted to prevent artefacts due to ERβ over-expression in the cell and to mimic what observed in primary breast tumors, where very high expression of this receptor has never been observed. As suitable antibodies for efficient immunoprecipitation of chromatin-bound ERβ are not available, the expressed proteins were tagged on either their C- (Ct-ERβ) or N-terminus (Nt-ERβ) with the TAP epitope. This approach allows to track tagged ERs in different cell compartments and to efficiently immunoprecipitate and purify them *in vitro *by Tandem Affinity Purification, to identify their molecular partners [5,33], and *ex vivo *in chromatin immunoprecipitation assays (see below). Preliminary tests were performed to verify whether the presence of the TAP moiety could influence intracellular redistribution of ERβ in response to 17β-estradiol (E2) and its ability either to *trans*-activate an estrogen-responsive reporter gene or to interfere with ERα activity on reporter gene transcription and BC cell proliferation. To this end, ER expression and nuclear translocation in response to E2 was determined in *wt *MCF-7, Ct-ERβ and Nt-ERβ cells by subcellular fractionation followed by SDS-PAGE and immunoblotting (Figure 1A). In absence of hormone a larger fraction of both ERs was found in the cytosol in all cases. Following estrogen stimulation, both receptors migrated to the nucleus, a crucial event to trigger target gene transcription *via *the genomic pathway of the estrogen signaling cascade. An antibody against α-tubulin was used as control, and the absence of this protein in the nuclear fractions indicates that they were indeed free from cytosolic contaminants. The ERβ-expressing clones selected for this study showed a ERβ/ERα ratio <2, as verified by immunoblotting analysis of the proteins in whole cell extracts and quantitative rtPCR of the corresponding RNAs [5, and data not shown]. To control that the presence of the TAP tag did not interfere with ERβ activity, ER-negative SKBR-3 BC cells were transiently transfected with expression vectors encoding *wt *ERβ, Ct-ERβ, Nt-ERβ, ERα (HEG0) or 'empty' vector (pSG5), as controls, and ERE-*tk*-luc [34], a reporter gene where luciferase expression is driven by an estrogen-responsive minimal promoter. Exposure of transiently transfected cells to E2 induced reporter gene activation in the presence of ERα, ERβ, Ct-ERβ or Nt-ERβ, with the activity of both tagged ERβ proteins slightly (15-20%) lower than that of *wt *ERβ (Figure 1B, left). We then tested whether the two recombinant forms of ERβ were able to interfere with target gene activation by the endogenous ERα resident in MCF-7 cells. To this end, *wt*, Ct-ERβ+ and Nt-ERβ+ cells were transfected with ERE-*tk*-luc and the response of the reporter gene to E2 was determined. As shown in Figure 1B (right), ERβ-expressing cells showed in all cases a marked (50-60%) reduction in reporter gene response to the hormone, when compared to *wt *MCF-7 cells, indicating that both tagged ERβs are able to interfere with the activity of endogenous ERα. Results show that cell lines stably expressing Ct-ERβ and Nt-ERβ display a marked reduction in proliferative response to the hormone, when compared to *wt *MCF-7 cells (Figure 1C), in agreement with the known effects of ERβ in ERα-positive cells [23,35-37]. Furthermore, comparative RNA expression profiling in exponentially growing Ct-ERβ and Nt-ERβ *vs wt *MCF-7 cells revealed extensive overlapping effects of the two tagged ERβ proteins on the activity of estrogen target genes [O. Paris *et al.*, manuscript in preparation and data not shown]. Taken together, these observations confirmed that both tag-ERβ expressing cell lines generated for this study show a well defined phenotype, with respect to the known activities of this ER subtype in BC cells, and are thus suitable to investigate the genomic bases of ERβ actions in this cell type. As we could not exclude that the presence of the TAP tag at either the N- or C- term of ERβ may specifically influence its activity on cellular targets or pathways different from those investigated above, all experiments reported in this study were performed in both Ct-ERβ and Nt-ERβ cells and the data were combined for analysis, with the aim to focus on the most significant and reproducible actions of ERβ independently from position of the tag in the receptor moiety.

### Effects of ERβ on the estrogen-responsive MCF-7 cell transcriptome

**Figure 2:**
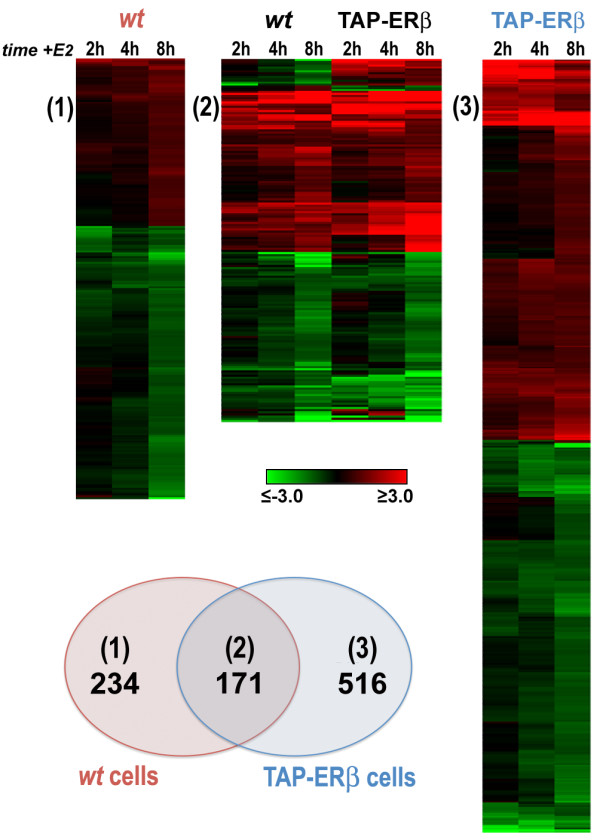
**Gene expression differences in absence or presence of ERβ**. *Top*: Heatmap summarizing the effects of ERβ expression of the estrogen responsive transcriptome of MCF-7 cells, showing changes in expression (log_2 _of the fold-change) of 921 transcripts after cell exposure to 10^-8^M E2 for the indicated times. Transcripts are grouped as follows: regulated only in *wt *MCF-7 cells (1), in both cell lines (2) and only in TAP-ERβ cells (3). *Bottom*: Venn diagram showing the numbers of differentially regulated by E2 in *wt *MCF-7 only (1), both cell lines (2) or ERβ expressing cells only (3).

Expression of ERβ is known to cause significant changes in the genomic response to estrogen in target cells. To identify the genes whose estrogen regulation in hormone-responsive BC cells is perturbed by ERβ, we performed gene expression profiling with microarrays in estrogen-starved, quiescent *wt *and TAP-ERβ+ (Ct-ERβ and Nt-ERβ) MCF-7 cells following stimulation with 10^-8^M E2. Total RNA was extracted from the three cell lines either before or after 2, 4 or 8 hrs stimulation, fluorescently labelled and analyzed on whole-genome microarrays. Samples extracted from the two ERβ-expressing cell lines were pooled before analysis, to reduce the impact of clone-specific differences and to focus on the most significant effects of ERβ, independent from tag position in the protein. Results obtained in these samples were then compared with those obtained under the same conditions in *wt *MCF-7 cells. This analysis yielded 921 transcripts differentially regulated by the hormone in ERβ+ *vs *ERβ- cells (Figure 2), including 234 mRNA whose regulation was detected only in *wt *MCF-7 cells, 516 regulated only in ERβ+ cells (see Venn diagram in Figure 2), 154 showing a similar pattern of response in both cell types (up- or down-regulated in all cases, although at different levels) and 17 showing opposite responses to the hormone in ERβ+ *vs *ERβ- cells (14 transcripts repressed in *wt *cells but activated in ERβ+ cells and 3 showing an opposite behaviour). The full list of these differentially regulated transcripts is reported, with relevant information, in Additional Table S1. Taken together, these results indicate that the presence of ERβ greatly influences the response of the MCF-7 cell genome to estrogen, by interfering with ERα-mediated hormonal regulation of 405 genes (Figure 2, left and central panels) and promoting *de novo *regulation of 516 genes (Figure 2, right panel). It should be noted that these analyses were performed with data obtained after 8 hrs of hormonal stimulation, a timing that allowed us to focus on early response genes, positioned upstream in the composite transcriptional cascade set in motion by the hormone in this cell type and more likely to include primary genomic targets of ERs [26]. It is thus possible that this analysis missed ERβ-responsive genes showing significant changes in expression only at later times after hormonal stimulation. However, analysis of the global effects of ERβ on gene expression in these same cells, performed as described above in cultures exponentially growing under continuous hormonal stimulus, suggests that the number of regulated transcripts identified here is rather close to the total number of genes targeted by this ER subtype in MCF-7 cells [O. Paris *et al.*, manuscript in preparation].

### Global mapping of ERα and ERβ binding to MCF-7 cell genome

**Figure 3:**
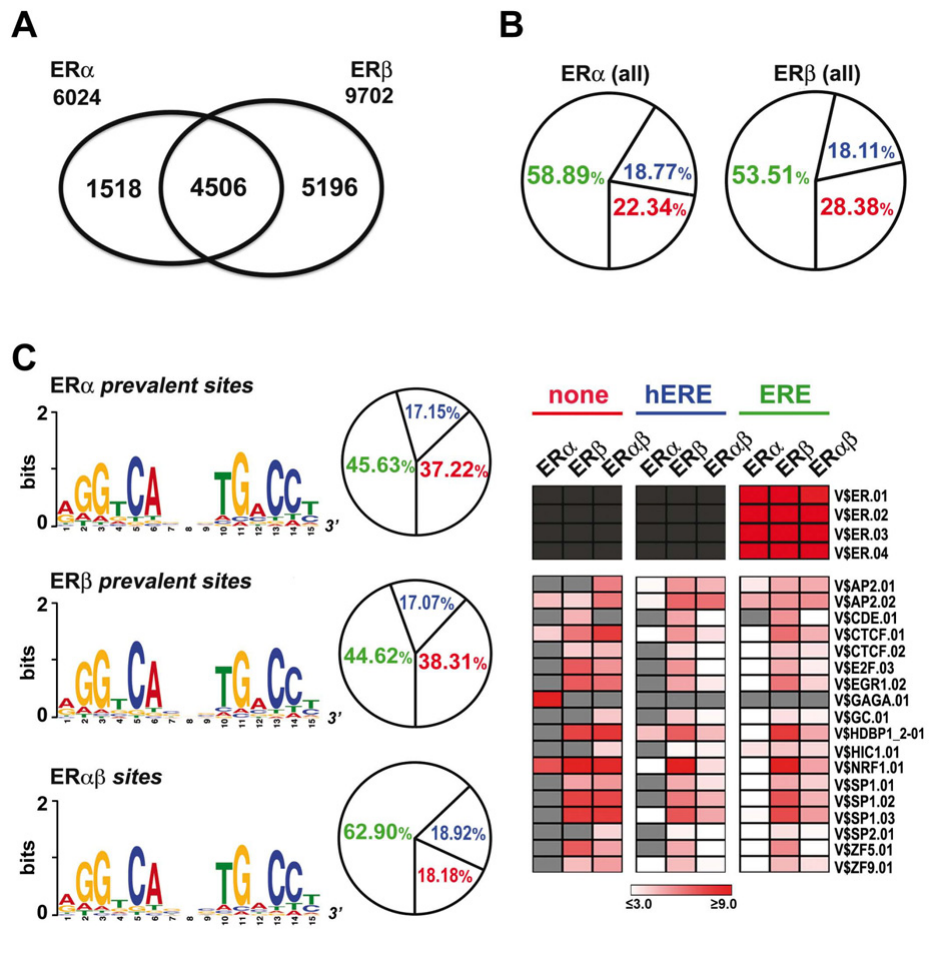
**Sequence analysis of ERα, ERβ and ERα+ERβ binding sites**. (A) Venn diagram showing a summary of ERα and ERβ binding sites identified in TAP-ERβ cells by ChIP-Seq. (B) Classification of ERα and β binding sites based on the presence of a perfectly or imperfectly palindromic Estrogen Response Element (ERE, green), an ERE hemipalindrome (hERE, blue) or no ERE (none, red). (C) ERE motif matrices identified in each of the three ER binding regions indicated (*left*), classification of the binding sites belonging to each region according to the presence of ERE (*center*) and grid summarizing the results of TFBS matrix enrichment (overrepresentation) analyses performed on the binding sites groups indicated (*right*). Z-Score cut-off was 3.0 and only TFBSs showing an over-representation score ≥4.0 in at least one of the ERE- (none) binding site groups. Light grey cells indicate a Z-Score <3.0 while dark grey cells indicate absence of the matrix.

The widespread effects of ERβ on MCF-7 cell transcriptome are likely to result from multiple effects of this receptor in the cells, including direct regulation of primary response genes *via *genomic or non genomic mechanisms, and indirect gene regulation events mediated by the products of primary genes. The primary ERβ target genes are most likely to comprise also master regulators of complex cellular responses to the receptor, mediating its effects on the biological and clinical phenotype of BC cells. To identify such primary genomic targets and investigate the mechanisms that allow their regulation by ERβ, a global analysis of *in vivo *binding of this receptor to the genome was carried out in TAP-ERβ cells by chromatin immunoprecipitation coupled to massively parallel sequencing (ChIP-Seq) [38], that allows detailed mapping of *in vivo *TF binding to the genome. In parallel, we studied ERα binding to the genome under the same conditions, to allow comparative analyses between the two ER subtypes. Replicate chromatin samples were prepared from both Ct-ERβ and Nt-ERβ cells before and after stimulation for 45 minutes with 10^-8^M E2 and DNA-bound proteins were immunoprecipitated either with antibodies against the N- and C-terminus of ERα, or with IgGs binding with high affinity the TAP moiety of tagged ERβ (see Methods). Preliminary testing on several known ERβ binding sites, including the promoter-near region of pS2/TFF1 gene [26], confirmed that the method selected to immunoprecipitate chromatin-bound Ct-ERβ and Nt-ERβ was efficient and specific (data not shown). The resulting DNAs were used to generate ChIP-Seq libraries for ERα and ERβ, respectively, that were then sequenced with the Illumina Genome Analyzer. The sequence tags obtained were then aligned to the human genome sequence and peaks enriched in the libraries generated after E2-treatment were identified using MACS (Model-based Analysis of ChIP-Seq) [39]. This led to the identification of 9702 binding sites for ERβ and 6024 sites for ERα, of which 4506 were shared by both receptors (Figure 3A), with an average False Discovery Rate (FDR) of 3%. The full list of these binding sites is available, with relevant information, in Additional Files 1 and 2. Interestingly, about half (4862) ERβ binding sites identified map within transcription units, mainly (3942 sites) in intronic regions. This distribution is maintained also in 424 ERβ-regulated transcription units (see below), where 966 ERβ binding sites located in the gene or within 10 kbps from it are distributed as follows: 154 in promoter regions, 51 in exons, 471 within one or more introns and the remaining either upstream of promoters (156) or downstream of the gene (134). In both cases the ERβ binding sites within genes did not show any preference with respect to exon or intron position nor for know intragenic regulatory elements (splice sites, polyadenylation sites, etc). It should be mentioned that the number of ERβ binding sites identified is significantly higher that those mapped in MCF-7 cells by ChIP-on-chips [30,31], possibly for technical differences due to ERβ expression levels in the different MCF-7 cell-derived clones used, in immunoprecipitation efficiency and/or in DNA analysis. Since only Zhao et al. [31] performed an unbiased search for ERβ binding with whole-genome chips, we could confront our results only with those reported in that study. This showed that 86% of high confidence ERβ sites described in that study appear also in our dataset. The binding sites identified here were then subjected to sequence analysis, searching first for the presence of EREs (Estrogen Receptor Elements), the characteristic ER binding signature (Figure 3B). This analysis revealed that in all three cases (i.e. ERβ, ERα and ERβ+ERα) a high percentage of sites displayed one or more imperfectly palindromic ERE (ERE+), with a slightly higher positivity in ERα sites (58.89 *vs *53.51%). As ERs have been shown to bind both *in vitro *and *in vivo *to PuGGTCA hemi-palindromes (hEREs), we searched the sequence of the remaining (ERE-) sites for perfect matches to this sequence. Results showed that almost half of them indeed contained one or more hEREs. The percentages of sites not carrying a known ER-binding element (ERE- and hERE-) were similar for both receptors (ERα: 22.34%, ERβ: 28.38%). We observed that ERα and ERβ binding sites were often found in close proximity to each other, a confounding factor when attempting to discern and analyze separately ER subtype-specific sites and target genes. This could be due to the limits of the ChIP-Seq technology or of the algorithm used for peak selection. To overcome this problem, and allow the identification of potential ER subtype-specific sites, we used a cartographic approach to group nearby binding sites that might be the result, at least in part, of shortfalls of the mapping methods applied. Each binding peak was thus elongated in both directions by 1000 bp and the overlapping ones obtained were merged into 8536 ERβ and 5371 ERα 'extended' binding regions. These regions were intersected to define ERα only, ERβ only or ERα+ERβ binding regions. In this way we could identify 1271 ERα-only and 4541 ERβ-only binding sites, comprised in these regions, none of which showed nearby binding of the other receptor. These were named: ER subtype 'prevalent' sites. The binding peak sequences included in each of the three regions obtained (ERα only, ERβ only or ERα+ERβ) were then re-analyzed for the presence of ERE or hERE elements. In this way we could observe that sites within the ERα+ERβ regions showed now a much higher percentage of ERE+ sequences (62.90%), respect to those present in the ERα-only or ERβ-only regions (45.63% and 44.62%, respectively, Figure 3C). Since all three types of sites showed almost identical proportions of hERE+, this result suggests that perfectly or imperfectly palindromic EREs are preferential binding sequences for ERα-ERβ heterodimers, while ERα and ERβ homodimers appear to be more flexible in DNA recognition. ERE+ sequences were then analyzed in more detail with MEME [40], to investigate if the three classes of sites identified showed any difference in the relative base composition of their respective ERE signatures. For each list of sequences, the most significant position-specific probability matrix generated by MEME was compared to the matrices present in the JASPAR transcription factor binding profile database [41], using the STAMP tool-kit for DNA motif comparison [42]. As shown in Figure 3C (left panels), this analysis revealed that the ERE matrices derived from the three types of binding regions identified (ERα selective, ERβ selective and ERα+ERβ) are identical and, as a consequence, that ERβ does not appear to display ERE variant selectivity.

We then examined the ERE- sequences to search for enriched binding motifs for other transcription factors that might play a role in ER binding to chromatin in the absence of canonical EREs. ERs are known to be able to bind chromatin indirectly, by physically interacting with DNA-bound TFs (tethering), including SP1 [43,44] or the AP1complex [31,45,46], for gene *trans*-regulation. TFBS enrichment respect to the whole genome was calculated thus in ERE- sites with using the RegionMiner tool [47] and only statistically significant (Z-score ≥3.0) and highly enriched (over-representation ≥4-fold) matrices were further considered. The results are summarized in the right panel of Figure 3C, showing for comparison the over-representation values scored in hERE+ and ERE+ sites by the same matrices selected in the ERE- sites. These numerical values, together with relevant information, are reported in Additional Table S2 [Additional file 3]. The enriched matrices found in the ERE- set of ERβ prevalent sites include V$SP1.01, V$SP1.02, V$SP1.03, V$SP2.01 and V$GC.01, all belonging to the GC-Box family targeted by SP1 and GCFC1 (GC-rich sequence DNA-binding) factors, V$CTCF.01 and V$CTCF.02, binding site matrices for the CCCTC-binding factor CTCF, that is a known transcriptional repressor of c-myc [48], V$NRF1.01, binding NRF1 (nuclear respiratory factor 1), a transcription factor that regulates the expression of nuclear-encoded mitochondrial genes [49], V$ZF5.01, for the POZ domain zinc finger protein ZF5, and V$ZNF9.01, recognized by the zinc finger proteins ZNF148, 202, 219 and 281. The majority of these TFs bind GC- and C-rich sequences that are structurally related to each other, suggesting the possibility that a significant portion of the sequence elements listed above might indeed be recognized by one or a limited number of TFs. On the other hand, the V$GAGA.01 matrix was specifically enriched only in the ERα prevalent ERE- binding sites. This sequence binds a transcription factor known to influence chromatin structure in *Drosophila *[50] and to bind throughout the genome [51], but nothing is known about physical or functional interactions of this factor with ERs or other nuclear receptors. The results of this analysis point to TFs that could act as partners of ERβ for binding to chromatin in the absence of canonical EREs. Interestingly, the majority of these same matrices were found enriched also in the ERβ binding sites comprising hEREs or EREs (Figure 3C), suggesting that one or more such DNA matrices might cooperate with ERβ for either DNA binding or gene *trans*-regulation. We performed a direct search for conserved motifs in the ERE- binding sites of ERβ with MEME [40], and the most significant position-specific probability matrices were compared to those present in the JASPAR TF binding profile database [41]. The results failed to provide any conclusive information, as each of several sequence motifs obtained with this analysis was found only in a small fraction of the binding sites analyzed.

### Identification of primary ERβ target genes

To identify the genes directly controlled by ERβ binding to the genome, and analyze the interplay between the two ERs in gene regulation, we combined the ChIP-Seq data with those relative to estrogen responsive genes differentially regulated by E2 in ERβ+ *vs *ERβ- MCF-7 cells under the same experimental conditions (Figure 2). Three sets of ER binding regions (defined as described in Methods) were used for this analysis: ERβ (8872, Set 1) and ERα (5558, Set 2) binding regions identified in TAP-ERβ cells, and all ERα binding regions identified so far in *wt *MCF-7 (17888, Set 3). The regions from Set 1 (ERβ in ERβ+ cells) were intersected with those from Set 2 (ERα in ERβ+ cells), to define which of them was binding both receptor subtypes ('heterodimer ERα+ERβ': 4186), only ERβ ('homodimer ERβ': 4686) or only ERα ('homodimer ERα': 1372) in TAP-ERβ cells. The 'homodimer ERβ' and the 'heterodimer ERα+ERβ' groups were further filtered against Set 3 binding regions (ERα sites detected in ERβ- cells), to identify the genomic sites recognized by ERβ, with or without ERα, but never by ERα alone. This allowed us to classify the sites comprised in these regions as follows: Class 1, including 2126 sites occupied by ERα in *wt *MCF-7 cells and by ERβ in TAP-ERβ cells (ERβ *vs *ERα competition); Class 2, showing 4340 sites where ERα can bind in *wt *MCF-7 cells and both receptors are detected in TAP-ERβ cells (ERβ+ERα); Class 3, with 2707 sites binding only ERβ and never, under any condition, ERα (ERβ specific); Class 4, comprising 529 sites where both receptors are found in TAP-ERβ cells but none in *wt *MCF-7 cells (ERβ+ERα specific); Class 5, including 617 sites where ERα binds only in TAP-ERβ but never in *wt *cells (ERα displacement); Class 6, composed of 773 sites that bind only ERα both in *wt *and TAP-ERβ cells (ERα specific). When combined with the results of the sequence analyses described above, this classification reveals that ERβ-specific *cis*-acting regulatory elements are unlikely to exist in the genome, as all evidence point to the fact that the two ER subtypes can recognize identical sequence features.

**Figure 4:**
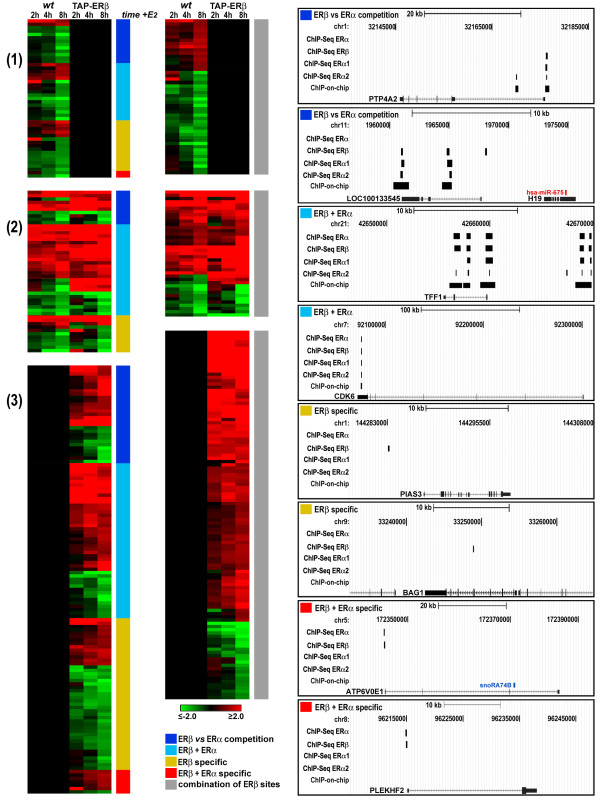
**Putative ERβ primary targets**. *Left*: Heatmap summarizing the effects of estrogen stimulation on 424 mRNAs encoded by genes showing one (*left*) or more (*right*) ERβ binding sites within 10 kb of the TU (primary response genes) transcriptome of MCF-7 cells, as changes in expression (log_2 _of the fold-change) after cell exposure to 10^-8^M E2 for the indicated times. Transcripts are grouped as follows: regulated only in *wt *MCF-7 cells (1), in both cell lines (2) and only in TAP-ERβ cells (3). Vertical bars to the right of each heatmap indicate the class of ERβ binding site present, as indicated in the legend. When a regulated gene showed multiple ERβ binding sites belonging to different classes it was included in a separate group, classified as 'combination of ERβ sites' (grey bar). *Right*: Genome Browser view of genomic loci representative of the different ERα and ERβ binding site categories identified. ChIP-Seq ERα and ChIP-Seq ERβ indicate sites identified in this study, ChIP-Seq ERα1, ChIP-Seq ERα2 and ChIP-on-chip indicates sites identified in MCF-7 cells by Cicatiello et al. [26], Fullwood et al. [27], and Hurtado et al. [28], respectively.

To identify among the genes differentially regulated by estrogen in ERβ+ *vs *ERβ- cells those representing direct targets for transcriptional regulation by DNA-bound ERβ in our cell model, we extracted from the list in Additional Table S1 [Additional file 3] the genes bearing one or more ERβ binding sites inside or within 10 kb of the TU, and termed them 'primary', to indicate that they are most likely to respond directly to the signal conveyed by the E2-activated receptor [26]. Of these 424 genes - listed in Additional Table S3 [Additional file 3], whose kinetics of response to E2 in *wt *and TAP-ERβ cells is shown in Figure 4, 52 show one ERβ site of Class 1 (ERβ *vs *ERα competition), 90 a site of Class 2 (ERβ+ERα), 71 a site of Class 3 (ERβ specific) and only 9 a Class 4 site (ERβ+ERα specific), while 202 showed multiple ERβ sites belonging to different classes and were thus classified accordingly (grey in Figure 4). In the right panels of Figure 4 are reported examples for each of the primary gene classes described above, showing the location of the receptor binding sites respect to the promoter and structural gene. It is worth mentioning that when the gene expression data from *wt *MCF-7 cells stimulated with E2 for 8hrs (Figure 2) were combined with information concerning ERα binding regions identified in *wt *MCF-7 cells under comparable conditions (Set 3 described above), 228 putative primary ERα target genes were identified -listed in Additional Table S4 [Additional file 3], 71% of which (163 genes) showed ERβ binding in hormone-stimulated ERβ+ cells. This result supports the view that the two ER subtypes tend to interact with the same targets in BC cells genome.

A functional analysis of the primary ERβ target genes identified here with Ontologizer [52] showed that most primary ERβ responsive genes are involved in key cellular processes, including control of cell proliferation, survival and differentiation status as well as cell motility, migration and adhesion, and can all greatly influence BC cell phenotype and response to estrogen - listed in Additional Table S5 [Additional file 4]. When GO analysis was performed on the ERα target gene set from *wt *MCF-7 cells (228 genes), results showed that the genes controlled directly by this ER subtype appear to be involved in the same cellular processes described above for ERβ -compare results reported in Additional Tables S5 and S6 [Additional files 4 and 5], providing a further indication of the significant overlapping between gene pathways targeted by ERβ and ERα in BC cells. Focusing on the genes differentially regulated by E2 in ERβ- *vs *ERβ+ cells known for their involvement in cell proliferation, we observed that many of them encode for transcription factors and other key proteins controlling large gene networks of cell division cycle and cell survival and, in general, cell proliferation and differentiation pathways. These include, in particular, CDK-6 (cyclin-dependent kinase 6), CEBPA (CCAAT/enhancer binding protein, alpha), DAB2 (Disabled homolog 2, mitogen-responsive phosphoprotein), HES-1 (Hairy and enhancer of split homologue 1), IGFBP-4 (Insulin-like growth factor binding protein 4), IRS-1 and -2 (Insulin receptor substrates 1 and 2), JAK-2 (Janus kinase 2), JunB, MITF (Microphthalmia-associated transcription factor), MYC, SLIT-2 (Slit homolog 2, *Drosophila*), SMARCA-2 (SWI/SNF related, matrix associated, actin dependent regulator of chromatin, subfamily a, member 2), SOX-9 (Sex determining region Y-box 9), TGFB-2 (Transforming growth factor beta 2), TGFBI/LCD-1 (transforming growth factor, beta-induced, 68 kDa), TGM-2 (Transglutaminase 2), TNS-3 (Tensin 3) and WISP-2 (WNT1 inducible signaling pathway protein 2). Interestingly, the role of all these genes in tumor cell proliferation and differentiation is known and an involvement in hormone-mediated BC cell responses to ERα has been reported for most of them, suggesting that discovery of an ERβ direct effect on transcription of these genes provides a new molecular framework to elucidate the anti-proliferative and differentiative effects of this receptor subtype in hormone-responsive cells.

Among the RNAs encoded in the genome, microRNAs (miRNAs) have emerged as master regulators of gene expression for their ability to influence mRNA concentration and activity by post-transcriptional mechanisms. Recent results highlighted the role of miRNA in BC cells response to estrogen [26,53-58] and, in addition, several lines of evidence indicate extensive miRNA deregulation in BC, including differential expression of miRNAs in normal *vs *transformed mammary epithelial cells [59-61]. For these reasons, we focused our attention on the TUs encoding pre-miRNAs, to test the possibility that ERβ binding sites might be located in close proximity of these genes. Results show that 52 miRNA-encoding loci (isolated or in genomic clusters) show one or more ERβ site within 10 kb from the pre-miR sequence of the host gene - listed in Additional Table S7 [Additional file 6]. Distribution of these sites among the ER binding Classes described above was comparable to what observed for primary genes. Interestingly, in several cases ERβ binds both up- and down-stream of the pre-miR locus, further suggesting that receptor docking might exert multiple regulatory actions on miRNA biogenesis.

We tested the hypothesis that the observed distribution of sites near the pre-miRNA loci occurred at random by applying a bootstrap approach. To this end, we repeatedly sampled 1000 times the same number of sites of the real ERβ binding set, with the same length distribution, a similar distribution among chromosomes but randomly selected coordinates. We then counted the number of pre-miRNA loci and the number of randomly generated sites found within 10 kb of each other and compared their distributions with that of the experimentally detected ones. The number of randomly generated sites within 10 kb of a pre-miRNA never reached the value detected experimentally, while the number of miRNA loci scoring an artificial site in close proximity was equal or above what measured only in 7.6% of the cases. These results can be explained also by the observation that in several cases ERβ binds both up- and down-stream of the pre-miR locus, a result that support a functional significance of this observation. In fact, the ratio between the number of ERβ binding sites and the number of pre-miRNA loci within 10 kb of each other is 1.5, while this varied between 0.5 and 1 for the randomly generated sets (data not shown). We thus conclude that although some of the ERβ sites detected in or near pre-miRNA loci may be the result of a random, non functional event, these is likely to represent rare events, as random distribution never reaches the enrichment level observed experimentally. Indeed, preliminary miRNA profiling analyses carried out in *wt *MCF-7 and TAP-ERβ cells indicate that mature miRNA expression undergo extensive deregulation in the presence of ERβ [O. Paris *et al.*, manuscript in preparation and data not shown], suggesting that at least some of the sites identified here might indeed be involved in ERβ-mediated regulation of miRNA biosynthesis in BC cells.

### ERβ binding to the mitochondrial genome

**Figure 5:**
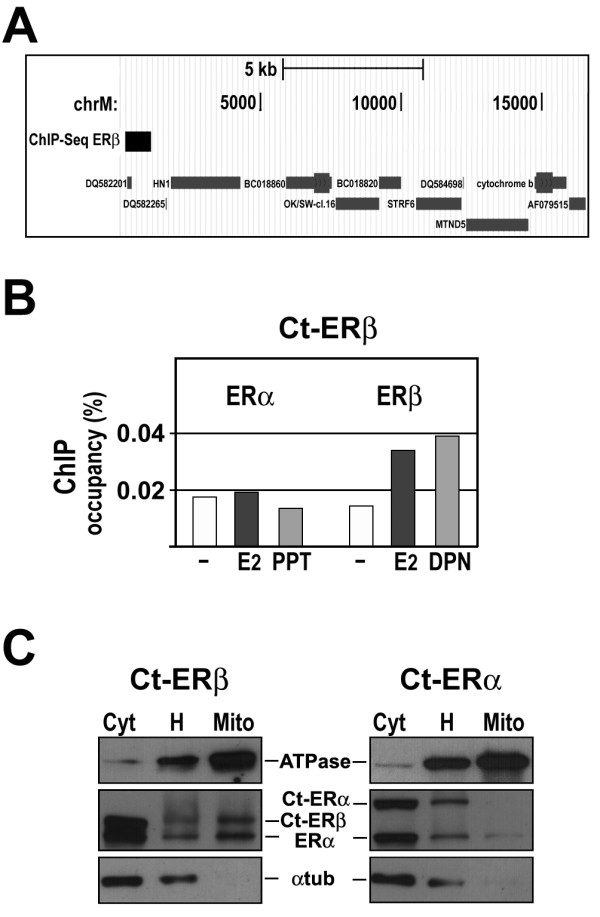
**Mitochondrial ER-beta binding sites**. (A) Genome Browser view of the ERβ binding site identified in mitochondrial genome. (B) Validation of ERβ binding to mitochondrial DNA by ChIP. Results shown are representative of duplicate analyses. E2: 10^-8^M 17β-estradiol; PPT: 10^-8^M 1,3,5-tris(4-hydroxyphenyl)-4-propyl-1H-pyrazole (selective ERα agonist); DPN: 10^-8^M 2,3-bis(4-hydroxyphenyl) propionitrile (ERβ agonist). (C) Western blot analysis of ERβ and/or ERα in purified mitochondria from Ct-ERβ and Ct-ERα [33] cells. Cyt: cytosol depleted of mitochondria, H: whole cell homogenate; Mito: purified mitochondrial fraction. ATPase is a mitochondrial marker and α-tubulin was included to determine the level cytosolic contaminants in 'Mito' samples.

Mitochondrial DNA (mtDNA) is usually overlooked in whole-genome ChIP-seq analyses, since identification of enriched peaks is more difficult here due to a much higher noise, consequence of the high and variable number of mtDNA copies in the cell. ERβ has been shown to localize in the mitochondria in different cell types [62] including human BC cells [63,64], and a role for estrogen in mitochondrial function, with implications on cell growth, has been established [65,66]. We thus analyzed the sequence reads that aligned on the mtDNA sequence with the same method used for whole genome data analysis, but applying a supplementary fold-intensity filter (see Methods) to deal with the higher background noise. This analysis revealed one ERβ binding site in proximity of the mtDNA D-loop, but no ERα binding sites either in *wt *MCF-7 or in TAP-ERβ cells (Figure 5A). Independent ChIP analysis confirmed this results, showing ERβ binding to this same mtDNA region upon activation by E2 or its selective agonist 2,3-bis(4-hydroxyphenyl) propionitrile (DPN) and lack of ERα binding (Figure 5B). Furthermore, the presence of ERβ in mitochondria of TAP-ERβ cells was confirmed biochemically, by western blotting (Figure 5C), an analysis that revealed also the presence of ERα in the organelle. When we analyzed the sequence of the ERβ mitochondrial site with MatInspector, we observed the presence of the matrix V$GATA1.06, bound by GATA1, a factor whose activity is strongly repressed by ERα [67], and V$HMGA.01, bound by HMGA1, a non-histone chromosomal protein that is highly overexpressed in cancer cells [68] and has been shown to interact with ERα and to enhance its binding to DNA [69].

## Conclusions

The results of this study indicate that *in vivo *ERβ can interact with hormone-responsive BC cell chromatin either alone or complexed with ERα, but in all cases the two receptors share the same genomic targets. An observation that is in agreement with the conclusions of previous studies based on analysis of ERα and ERβ heterodimerization and binding to the ERE [9,70-72]. When both ER subtypes are expressed in the same cell, the main action of ERβ in the genome is thus achieved in combination with ERα, by either heterodimerization or competition for binding to the same target sites in chromatin. Based on these observations, we propose that in hormone-responsive BC the final cellular response to estrogen is likely to depend upon the relative concentration of the two ERs in the cell, their activation status and DNA binding kinetics and the presence of other factors influencing their respective functions.

## Methods

### Plasmid preparation, cell lines, cell culture and stable transfections

Different BC cell lines were used: MCF-7 TET Off (ER-alpha positive; ATCC, Cat No. HTB-22) and SKBR3 (ER-alpha negative; ATCC, Cat No. HTB-30). MCF-7 TET Off cells (described here as *wt *or ERβ-) were used to produce stable clones expressing ERβ tagged with TAP-tag respectively at the C-term and at the N-term (Ct-ERβ and Nt-ERβ) or C-tagged ERα (Ct-ERα), as previously described [5,33]. All were grown in Dulbecco's modified Eagle's medium (DMEM), supplemented with 10% fetal bovine serum (FBS) (both from Invitrogen), 100 U/ml penicillin, 100 mg/ml streptomycin, 250 ng/ml Amfotericin-B, 50 μg/ml G418 (normal growing condition). For estrogen starvation, cells were plated at 40% confluence in steroid-free medium (phenol red-free DMEM medium, with 5% fetal bovine serum pre-treated with dextran-coated charcoal and antibiotics) and maintained for 5 days with replacement of fresh medium before stimulation with 10^-8^M 17β-estradiol (E2).

### Preparation of cell extracts, mitochondria isolation and immunoblotting analyses

Cells starved in 100-mm dishes were stimulated for 45 minutes, harvested in cold PBS and collected by centrifugation at 1000 × g. The cell pellets were then resuspended in three volumes of Hypotonic Buffer (HB) (20 mM HEPES pH 7.4, 5 mM Sodium Fluoride, 10 μM Sodium Molybdate, 0.1 mM EDTA, 1 mM dithiothreitol, 1 mM protease inhibitors, 1 mM Phenylmethyl-Sulfonyl Fluoride). Cells were then incubated on ice for 15 min and 0.5% Nonidet P-40 followed by spinning 30 sec at 4°C at 13000 × g. Supernatants were recovered and clarified at 13,000 × g for 15 min at 4°C while pellets were resuspended in hypotonic buffer, stratified on 25% sucrose-HB solution and centrifuged at 6000 × g for 15 min at 4°C. The resulting pellets were then resuspended in one volume of Nuclear Lysis Buffer [73] containing 800 mM NaCl, incubated for 30 min at 4°C with gentle shaking and centrifuged for 15 min at 4°C at 13000 × g. The supernatant fraction was recovered.

Mitochondria were isolated from 20 × 10^6 ^Ct-ERβ or Ct-ERα cells (in 150 mm culture dishes) as described [74], with minor modifications. All steps during mitochondria isolation were performed at 4°C, cells were washed twice in PBS, scraped, and centrifuged at 290 × g, 5 minutes. The samples were resuspended in buffer A (250 mM Sucrose, 50 mM Tris-HCl, 2 mM EGTA), homogenized in Glass-Teflon Potter homogenizer and centrifuged at 600 × g for 3 min, then the supernatants were re-centrifuged at the same speed. Mitochondria were pelleted by centrifugation at 10400 × g for 10 min, resuspended in buffer A and centrifuged again at 5300 × g for 10 min, in order to eliminate microsomal and cytosolic contamination. The samples were dissolved in buffer A and centrifuged at 1500 × g for 4 min and then pelleted again at 9600 × g for 10 minutes. The final pellet was resuspended in Buffer B (50 mM Tris pH 7.5, 150 mM NaCl, 1% Triton X-100, 0.1% SDS, 1% Na Deoxycholate, 1 mM PMSF, 1X Protease Inhibitor) and incubated on ice for 20 min to extract mitochondrial proteins. A small portion of sample was collected after homogenization and processed to obtain the samples corresponding to homogenate and cytosol. An equivalent protein amount was fractionated by SDS-PAGE on Mini Protean Precast polyacrylamide gels 4-20% from Biorad.

Homogenate, cytosol, nuclear or mitochondrial extracts from equivalent cell number were fractionated by SDS-PAGE. Immunoblot analysis was performed using the following primary antibodies: Ct-ERα (HC-20; sc-543) from Santa Cruz Biotechnology, Inc., TAP tag (CAB1001) from Open Biosystems, α-Tubulin (T 6199) from Sigma-Aldrich, ATPase B (ab14730) from Abcam. Peroxidase-labelled anti-rabbit or -mouse immunoglobulin antisera were used according to the manufacturer's instructions (Amersham Italia).

### Transient Transfections and Luciferase Assay

Wild type MCF-7, Ct-ERβ and Nt-ERβ clones were starved for 5 days in estrogen-free medium. Then 5 × 10^5 ^cells/dish were seeded in 60 mm culture dishes and transfected by using 25 μg/dish polyethylenimine (Polysciences, Inc.) with 2.5 μg/dish DNA, including 300 ng ERE-tk-Luc [75], 500 ng pSG-Δ2-NLS-LacZ vector [76], co-transfected as an internal control for transfer efficiency, and carrier DNA (Bluescribe M13+). At 48 hrs after transfection, cells were treated for 24 hrs with either vehicle (EtOH) or E2 (10^-8^M).

SKBR3 cells were grown to 60-70% confluence and transfected with Lipofectamine 2000 reagent (Invitrogen) and OPTI-MEM (Invitrogen) according to the manufacturer's instructions. The plasmids used were pSG5-ERβ, encoding full-length ERβ (ERβ1), pSG-HEGO, encoding the full-length ERα and the corresponding pSG5 empty vector (Stratagene), pUSE-C-TAP-ERβ and pUSE-N-TAP-ERβ, encoding full-length ERβ tagged, respectively, at the C-term and at the N-term [5], ERE-*tk*-Luc and pSG-Δ2-NLS-LacZ. Six hours after transfection, the medium was changed and 24 hrs later cells were stimulated as described above for 24 hrs. At the end of treatment, cells were washed with cold PBS and lysed in 100 μl lysis buffer (Promega). Luciferase activity was measured in extracts using the Luciferase Assay Reagent (Promega), according to the manufacturer's instructions, and values were expressed as relative light units normalized to the β-galactosidase activity or to the protein concentrations measured using the Bradford technique. For each condition, average luciferase activity was calculated from the data obtained from three independent dishes.

### Cell Cycle Analysis

Estrogen-deprivation was always controlled by cell cycle analysis as follows. Cells (1.5 × 10^5 ^cells/dish) were starved in 60 mm culture dishes, stimulated for 27 hrs and collected in PBS containing 50 μg/ml propidium iodide, 0.1% (v/v) sodium citrate, 0.1% (v/v) Nonidet P-40. Cell samples were incubated in the dark for at least 15 min at room temperature, or overnight at 4°C, and analyzed by a FACScalibur flow cytometer using the CellQuest software package (BD Biosciences), according to standard protocols suggested by the manufacturer [77,78]. Data analysis was performed with Modfit software (Verity Software, Topsham). Values were plotted as increasing of S-phase respect to unstimulated controls. Results showed were obtained from two independent experiments.

### Cell Proliferation Assay

Hormone-starved cells (3000/well) were seeded in 96-well dishes. After 12 hrs, medium was changed to include the indicated compounds. After appropriate stimulation, cells were washed in phosphate-buffered saline (PBS) and fixed with 12.5% glutaraldehyde for 20 min at room temperature, followed by washing with distilled water, incubation with 0.05% methylene blue for 30 min, rinsing and incubation with 0.33 M HCl for 18 hrs. Absorption was measured at 620 nm.

### RNA purification

Total RNA was extracted from *wt *MCF-7, Ct-ERβ and Nt-ERβ clones, generated as described above, using the standard RNA Extraction method with TRIzol (Invitrogen) method, as described previously [79,80]. Cells were estrogen-deprived and total RNA was extracted before or at the indicated times after stimulation with 10^-8^M 17β-estradiol (+E2) or ethanol vehicle.

In each case RNA derived from two independent experiments performed in duplicate was used. Before use, RNA concentration in each sample was assayed with a ND-1000 spectrophotometer (NanoDrop) and its quality assessed with the Agilent 2100 Bioanalyzer with Agilent RNA 6000 nanokit (Agilent Technologies).

### Microarray analyses

Total RNA extracted from Ct-ERβ and Nt-ERβ cells were pooled. For mRNA expression profiling, 500 ng total RNA were reverse transcribed, as described previously [26,81] and used for synthesis of cDNA and biotinylated cRNA according to the Illumina TotalPrep RNA Amplification Kit (Ambion, Cat. n. IL1791) protocol. For each sample, 750 ng of cRNA were hybridized for 18 hrs at 55°C on Illumina HumanHT-12 v3.0 BeadChips, containing 48,804 probes (Illumina Inc.), according to the manufacturer's protocol and subsequently scanned with the Illumina BeadArray Reader 500. Data analyses were performed with GenomeStudio software version 2009 (Illumina Inc.), by comparing all values obtained at each time point against the 0 hrs values. Data was normalized with the quantile normalization algorithm, and genes were considered as detected if the detection p-value was lower than 0.01. Statistical significance was calculated with the Illumina DiffScore, a proprietary algorithm that uses the bead standard deviation to build an error model. Only genes with a DiffScore ≤-40 and ≥40, corresponding to a p-value of 0.0001, were considered as statistical significant.

### Chromatin Immunoprecipitation

Ct-ERβ and Nt-ERβ cells were hormone-deprived for 4 days and chromatin was extracted in several replicates before (-E2) and after stimulation for 45 minutes with 10^-8^M 17β-estradiol (+E2) or, where indicated, with the 10^-8^M selective ERα agonist 1,3,5-tris(4-hydroxyphenyl)-4-propyl-1H-pyrazole (PPT) or 10^-8^M ERβ agonist 2,3-bis(4-hydroxyphenyl) propionitrile (DPN), from Tocris Cookson.

Chromatin was prepared with the Millipore/Upstate Chromatin Immunoprecipitation (ChIP) Assay Kit (Millipore) according to the instruction provided by the producer, using a variation of the protocol described at the Upstate website. For each assay, a total of 5 × 10^6 ^cells were fixed with 1% formaldeyde for 10 min at room temperature, the reaction was then stopped by adding glycine at final concentration of 0.125 M. Fixed cells were washed twice with ice-cold PBS, harvested by scraping, centrifuged and the cell pellets were re-suspended in SDS lysis buffer. Samples were sonicated with a Diagenode Bioruptor (Diagenode) for 12 cycles of 30 sec at high power, centrifuged at 12500 xg for 15 minutes and diluted 8-fold in ChIP dilution buffer. After removing an aliquot (whole-cell extract input), the chromatin sample was divided in three aliquots, that were incubated at 4°C overnight with antibodies against either the C-term (HC-20, from Santa Cruz Biotechnology) or N-term (anti-Estrogen Receptor 18-32, from SigmaAldrich) of human ERα or with IgG Sepharose 6 fast Flow (GE Healthcare Bio-Science AB) for TAP-ERβ [5]. As control, an aliquot of the same chromatins were processed in the same way but Abs or IgGs were omitted form the reaction. The samples were then precipitated by binding to protein-A Agarose/Salmon Sperm DNA beads (Millipore), for ERα, or as such for to ERβ. The beads were washed sequentially with 'low-salt immune complex wash buffer, 'high salt immune complex wash buffer, 'LiCl immune complex wash buffer' and TE buffer, before elution in Elution buffer by ON incubation at 65°C and treatment with Proteinase K. DNA was purified from immunoprecipitated (IPP) chromatin by extraction with phenol:chloroform:isoamyl alcohol (25:24:1) and ethanol precipitation according to standard procedures. DNA pellets were dissolved in nuclease-free water and kept frozen before further use.

Primers for ChIP-QPCR validation of the mitochondrial genome ERβ binding site were the following: 5'-GATCACAGGTCTATCACCCTATTAACC (forward) and 5'-CAGCGTCTCGCAATGCTATC (reverse).

### Samples preparation for ChIP-Seq

DNAs from Ct-ERβ and Nt-ERβ cells treated with E2 were pooled together to generate an ERβ +E2 sample and the same was done for DNAs from hormone-starved cells (ERβ -E2 sample). Similarly, IPP DNAs obtained with anti-C-term and anti-N-term ERα Abs were pooled together to generate ERα +E2 and ERα -E2 samples. About 20 ng of ChIP DNA was purified using the MinElute PCR Purification Kit (QIAGEN, Italy), with a recovery of 55-70%, as assessed with the Quant-IT DNA Assay Kit-High Sensitivity and a Qubit Fluorometer (Invitrogen). Preparation of IPP DNA libraries for massively parallel sequencing was performed from 10 ng purified DNA according to the Illumina ChIP-Seq sample preparation kit protocol (Illumina Inc.). Libraries were sequenced with the Illumina Cluster Station and Genome Analyzer II according to manufacturer's instructions.

### ChIP-Seq data analysis

The sequence tags generated by massively parallel sequencing were aligned on the human genome (hg18) with the software ELAND, allowing up to 2 mismatches. Enriched regions from ERα +E2 and ERβ +E2 samples were compared with the same from ERα -E2 and ERβ -E2 samples, respectively. The enriched ChIP-Seq peaks were identified using MACS (Model-based Analysis of ChIP-Seq) version 1.3.7.1 [39], with standard parameters (p-value cut-off of 1e-5, mfold of 32). For mtDNA we further filtered out sites with tag density below 0.5 (N/l; N = number of tags, l = length of site)

### Computational searches for ERE sequences

The ERE binding motif was searched in binding sites with the MatInspector application [82], a part of GenomatixSuite software (Genomatix Software GmbH, Germany). The matrices ER.01, ER.02, ER.03 and ER.04 (Genomatix Matrix Library 8.2), were used with a core similarity threshold of 0.75 and a matrix similarity threshold of Optimal -0.02. The sequences bearing a match of any of the four matrices were termed ERE+ sequences. On the remaining sequences, the hemi-palindromic ERE motif was searched with the same application, by defining a custom half ERE matrix PuGGTCA (hERE). The remaining sequences were classified as ERE- and were scanned for other TF binding sites motifs contained in the Genomatix Matrix Library using the standard parameters, as described previously [26].

### TFBS over-representation analysis

ERE- sequences were analyzed to search for known TF binding sites (TFBSs) that were enriched (over-represented) with respect to their relative frequency in the whole human genome. This analysis was performed with the RegionMiner application of the Genomatix software suite [47]. The software automatically searches for all TFBS matches present in the submitted sequence list and calculates the over-representation value of the actual number of matches over the expected value based on its frequency in the reference set (genome or promoters) for each matrix. It reports also the significance of the over-representation ratio, expressed as Z-scores [83]. Enrichment values with a Z-score <3.0 were not considered further. A filter was applied also on the over-representation values, depending upon the range of values set for each analysis, to highlight only the stronger associations. Results are shown as heatmaps representing over-representation values, generated with MeV software [84].

### Classification of ERβ binding sites and identification of primary ERβ responsive genes by combining ChIP-Seq and expression profiling data

Three sets of ER binding sites were taken in consideration. The first and second comprised, respectively, the ERβ and ERα sites mapped in TAP-ERβ cells and the third (named 'MCF-7 ERα') included all ERα binding sites identified in *wt *MCF-7 cells by ChIP-Seq [26,27] and/or by ChIP-on-chip [28]. First of all, the ER binding sites from ChIP-Seq analyses were elongated in both directions by 1000 bp. Subsequently, using UCSC Table Browser [85], for each of the first two sets the extended sites overlapping with each other were merged in ERβ or ERα binding 'regions', respectively. For the third set (*wt *MCF-7 ERα), the extended ChIP-Seq sites and the ChIP-on-chip sites overlapping with each other in the genome were all merged to generate unique MCF-7 ERα binding 'regions'. To identify putative primary ERβ responsive genes, the TUs corresponding to RNAs differentially regulated by E2 in ERβ+ *vs *ERβ- cells that showed one or more ERβ binding region inside or within 10 kb were extracted using UCSC Table Browser, as described previously [26].

### Gene Ontology analysis

To identify Biological Process GO terms statistically overrepresented in our regulated gene lists, we used Ontologizer 2.0, a tool for GO term enrichment analysis of genes derived from an experiment [52]. We identified enriched terms in primary ERβ or ERα target genes against all genes expressed (detected) in the cell lines under study, set as background of the analysis, with a *p *value threshold of 0.01.

### Microarray and ChIP-Seq data accession numbers

The microarray and ChIP-Seq data have been deposited in the Array Express database ( HYPERLINK "http://www.ebi.ac.uk/arrayexpress" http://www.ebi.ac.uk/arrayexpress) with Accession Numbers E-TABM-1051 and E-MTAB-345, respectively.

## Competing interests

The authors declare that they have no competing interests.

## Authors' contributions

OMVG, MM, GG, MR, LC, MRDF, LF, GN, MFP, OP and RT participated in the conception and design of the study, performed *in vivo *and *in vitro *experimental work (OP, GN, MFP, RT), statistical and *in silico *data analyses (OMVG, MM, GG, MRDF) and participated in drafting the manuscript. OP, LC and MR prepared and tested the sequencing libraries. LC, LF and MR carried out the microarray experiments and participated in data analyses. SL, GPS and VB participated in the conception and design of the study and performed massively parallel sequencing. AW coordinated the project, participated in conception and design of the study and participated in drafting and finalization of the manuscript. All authors read and approved the final manuscript.

## Supplementary Material

Additional File 1 **Grober_ER-alpha_Binding_Sites ERα binding sites**. The UCSC genome BED formatted file lists the chromosome, start coordinate, stop coordinate and identifier of the ERα binding sites. Click here for file

Additional File 2 **Grober_ER-beta_Binding_Sites ERβ binding sites**. The UCSC genome BED formatted lists the chromosome, start coordinate, stop coordinate and identifier of the ERβ binding sites. Click here for file

Additional File 3 **Sheet 1: *Additional Table S1 *Differentially Regulated Genes**. Overview of the 921 genes differentially regulated by E2 in ERβ+ *vs *ERβ- cells, containing the following additional information: gene set membership, symbol, Entrez ID, gene name and expression values (fold-change). Sheet 2: *Additional Table S2 *TFBS enrichment matrix. The worksheet shows the over-representation values for the TF binding matrices from ERE- binding sites. Sheet 3: *Additional Table S3 *Primary ERβ target genes, showing ERβ binding sites within 10 kb of the TU. Overview of the 424 putative ERβ primary gene targets containing the following additional information: gene set membership, category of ERβ binding sites, Symbol, Entrez ID, Gene Name, TU Coordinates, ERα Binding Sites Coordinates and ERβ Binding Sites Coordinates. Sheet 4: *Additional Table S4 *Primary ERα target genes, showing ERα binding sites within 10 kb of the TU in ERβ- cells. Overview of the 228 putative ERα primary gene targets containing the following additional information: Symbol, Entrez ID, Gene Name, TU Coordinates. Click here for file

Additional File 4 **GO analysis of primary ERβ target genes**. Containing the following information: Biological process, Gene Ontology term, Name, Count in total GO population, Count in selected genes, % genes and p-value. Click here for file

Additional File 5 **GO analysis of primary ERα target genes**. Containing the following information: Biological process, Gene Ontology term, Name, Count in total GO population, Count in selected genes, % genes and p-value. Click here for file

Additional File 6 **ERβ binding sites in proximity of miRNA loci**. Containing the following information: miRNA name, ID of ER-β binding site upstream, Distance from the closest ERβ binding site upstream, ID of ER-β binding site downstream and Distance from the closest ERβ binding site downstream. Click here for file
