# Supplementary material for: Global analysis of estrogen receptor beta binding to breast cancer cell genome reveals an extensive interplay with estrogen receptor alpha for target gene regulation
Source: BMC Genomics. 2011 Jan 14;12:36. doi: 10.1186/1471-2164-12-36 (PMC3025958; doi:10.1186/1471-2164-12-36)
Supplement: Additional File 4 — GO analysis of primary ERβ target genes. Containing the following information: Biological process, Gene Ontology term, Name, Count in total GO population, Count in selected genes, % genes and p-value. [file 1471-2164-12-36-S4.DOC]

### **Additional Table 5. GO analysis of primary ERβ** target genes

| ***Biological process*** | **Gene Ontology term** | **Name** | **Count in total GO population** | **Count in selected genes** | **% genes** | ***p*-value** |
| --- | --- | --- | --- | --- | --- | --- |
| *Cell proliferation* | GO:0042127 | regulation of cell proliferation | 425 | 28 | 6.6 | 0.0096 |
|  | GO:0000212 | meiotic spindle organization | 4 | 1 | 25 | 0.0000 |
|  | GO:0051295 | establishment of meiotic spindle localization | 1 | 1 | 100 | 0.0001 |
| *Cell death* | GO:0043069 | negative regulation of programmed cell death | 238 | 20 | 8.4 | 0.0020 |
|  | GO:0043066 | negative regulation of apoptosis | 236 | 19 | 8.1 | 0.0041 |
| *Regulation of transcription* | GO:0010552 | positive regulation of gene-specific transcription from RNA polymerase II promoter | 53 | 7 | 13.2 | 0.0058 |
| *Response to stimuli* | GO:0006950 | response to stress | 933 | 57 | 6.1 | 0.0015 |
|  | GO:0009725 | response to hormone stimulus | 238 | 20 | 8.4 | 0.0020 |
|  | GO:0001666 | response to hypoxia | 84 | 12 | 14.3 | 0.0002 |
| *Signaling* | GO:0035556 | intracellular signal transduction | 672 | 41 | 6.1 | 0.0072 |
|  | GO:0007166 | cell surface receptor linked signaling pathway | 618 | 55 | 8.9 | 0.0000 |
|  | GO:0007267 | cell-cell signaling | 255 | 23 | 9 | 0.0003 |
|  | GO:0007186 | G-protein coupled receptor protein signaling pathway | 149 | 18 | 12.1 | 0.0000 |
|  | GO:0035467 | negative regulation of signaling pathway | 140 | 13 | 9.3 | 0.0052 |
|  | GO:0007219 | Notch signaling pathway | 32 | 5 | 15.6 | 0.0094 |
|  | GO:0010863 | positive regulation of phospholipase C activity | 15 | 4 | 26.7 | 0.0027 |
|  | GO:0046627 | negative regulation of insulin receptor signaling pathway | 14 | 4 | 28.6 | 0.0021 |
|  | GO:0014065 | phosphoinositide 3-kinase cascade | 14 | 4 | 28.6 | 0.0021 |
|  | GO:0070304 | positive regulation of stress-activated protein kinase signaling cascade | 10 | 3 | 30 | 0.0068 |
|  | GO:0048016 | inositol phosphate-mediated signaling | 3 | 2 | 66.7 | 0.0050 |
|  | GO:0043552 | positive regulation of phosphoinositide 3-kinase activity | 3 | 2 | 66.7 | 0.0050 |
| *Inflammatory response* | GO:0002526 | acute inflammatory response | 21 | 5 | 23.8 | 0.0014 |
|  | GO:0050729 | positive regulation of inflammatory response | 11 | 3 | 27.3 | 0.0090 |
|  | GO:0032637 | interleukin-8 production | 8 | 3 | 37.5 | 0.0034 |
| *Development and differentiation* | GO:0045595 | regulation of cell differentiation | 280 | 21 | 7.5 | 0.0059 |
|  | GO:0060429 | epithelium development | 171 | 17 | 9.9 | 0.0007 |
|  | GO:0021700 | developmental maturation | 50 | 7 | 14 | 0.0042 |
|  | GO:0002064 | epithelial cell development | 17 | 4 | 23.5 | 0.0044 |
| *Cell motility and adhesion* | GO:0007155 | cell adhesion | 337 | 32 | 9.5 | 0.0000 |
|  | GO:0048870 | cell motility | 229 | 26 | 11.4 | 0.0000 |
|  | GO:0016477 | cell migration | 221 | 26 | 11.8 | 0.0000 |
|  | GO:0030335 | positive regulation of cell migration | 60 | 8 | 13.3 | 0.0031 |
|  | GO:0050900 | leukocyte migration | 36 | 6 | 16.7 | 0.0032 |
|  | GO:0016337 | cell-cell adhesion | 130 | 15 | 11.5 | 0.0003 |
|  | GO:0033630 | positive regulation of cell adhesion mediated by integrin | 3 | 2 | 66.7 | 0.0050 |
| *Ion homeostasis* | GO:0055065 | metal ion homeostasis | 75 | 13 | 17.3 | 0.0000 |
|  | GO:0055074 | calcium ion homeostasis | 72 | 12 | 16.7 | 0.0000 |
|  | GO:0055067 | monovalent inorganic cation homeostasis | 26 | 6 | 23.1 | 0.0005 |
| *Metabolism* | GO:0006629 | lipid metabolic process | 491 | 33 | 6.7 | 0.0038 |
|  | GO:0016051 | carbohydrate biosynthetic process | 82 | 9 | 11 | 0.0065 |
|  | GO:0042445 | hormone metabolic process | 53 | 7 | 13.2 | 0.0058 |
|  | GO:0009187 | cyclic nucleotide metabolic process | 52 | 7 | 13.5 | 0.0052 |
|  | GO:0042219 | cellular amino acid derivative catabolic process | 11 | 3 | 27.3 | 0.0090 |
|  | GO:0070873 | regulation of glycogen metabolic process | 10 | 3 | 30 | 0.0068 |
|  | GO:0032000 | positive regulation of fatty acid beta-oxidation | 4 | 2 | 50 | 0.0097 |
|  | GO:0030818 | negative regulation of cAMP biosynthetic process | 3 | 2 | 66.7 | 0.0050 |
| *Transport and localization* | GO:0070201 | regulation of establishment of protein localization | 81 | 9 | 11.1 | 0.0060 |
|  | GO:0008643 | carbohydrate transport | 49 | 7 | 14.3 | 0.0037 |
|  | GO:0043269 | regulation of ion transport | 38 | 6 | 15.8 | 0.0043 |
|  | GO:0015749 | monosaccharide transport | 33 | 6 | 18.2 | 0.0021 |
| *Hormone secretion* | GO:0046887 | positive regulation of hormone secretion | 20 | 4 | 20 | 0.0082 |
