# Supplementary material for: Global analysis of estrogen receptor beta binding to breast cancer cell genome reveals an extensive interplay with estrogen receptor alpha for target gene regulation
Source: BMC Genomics. 2011 Jan 14;12:36. doi: 10.1186/1471-2164-12-36 (PMC3025958; doi:10.1186/1471-2164-12-36)
Supplement: Additional File 5 — GO analysis of primary ERα target genes. Containing the following information: Biological process, Gene Ontology term, Name, Count in total GO population, Count in selected genes, % genes and p-value. [file 1471-2164-12-36-S5.DOC]

### **Additional Table 6. GO analysis of primary ERα target genes**

| ***Biological process*** | **Gene Ontology term** | **Name** | **Count in total GO population** | **Count in selected genes** | **% genes** | ***p*-value** |
| --- | --- | --- | --- | --- | --- | --- |
|  | GO:0008283 | cell proliferation | 573 | 25 | 4.4 | 0,0025 |
| *Cell proliferation* | GO:0042531 | positive regulation of tyrosine phosphorylation of STAT protein | 5 | 2 | 40 | 0.0054 |
|  | GO:0042517 | positive regulation of tyrosine phosphorylation of Stat3 protein | 4 | 2 | 50 | 0.0033 |
| *Cell death* | GO:0043067 | regulation of programmed cell death | 505 | 22 | 4.4 | 0.0046 |
|  | GO:0042981 | regulation of apoptosis | 503 | 22 | 4.4 | 0.0044 |
| *Response to stimuli* | GO:0048545 | response to steroid hormone stimulus | 103 | 8 | 7.8 | 0.0032 |
|  | GO:0032355 | response to estradiol stimulus | 34 | 4 | 11.8 | 0.0084 |
|  | GO:0010039 | response to iron ion | 10 | 3 | 30 | 0.0014 |
| *Signaling* | GO:0007166 | cell surface receptor linked signaling pathway | 578 | 34 | 5.9 | 0.0000 |
|  | GO:0007167 | enzyme linked receptor protein signaling pathway | 222 | 14 | 6.3 | 0.0009 |
|  | GO:0007267 | cell-cell signaling | 239 | 13 | 5.4 | 0.0048 |
|  | GO:0007186 | G-protein coupled receptor protein signaling pathway | 137 | 11 | 8 | 0.0004 |
|  | GO:0046427 | positive regulation of JAK-STAT cascade | 6 | 2 | 33.3 | 0.0080 |
|  | GO:0043406 | positive regulation of MAP kinase activity | 46 | 5 | 10.9 | 0.0046 |
|  | GO:0048016 | inositol phosphate-mediated signaling | 2 | 2 | 100 | 0.0006 |
| *Inflammatory response* | GO:0006954 | inflammatory response | 112 | 8 | 7.14 | 0.0053 |
|  | GO:0032613 | interleukin-10 production | 6 | 2 | 33.3 | 0.0080 |
|  | GO:0001568 | blood vessel development | 142 | 10 | 7 | 0,0021 |
|  | GO:0001525 | angiogenesis | 97 | 8 | 8.2 | 0,0022 |
| *Development and differentiation* | GO:0048639 | positive regulation of developmental growth | 7 | 3 | 42.8 | 0.0004 |
|  | GO:0045597 | positive regulation of cell differentiation | 117 | 9 | 7.6 | 0.0019 |
| *Cell motility and adhesion* | GO:0016337 | cell-cell adhesion | 119 | 10 | 8.4 | 0.0005 |
|  | GO:0048870 | cell motility | 218 | 14 | 6.4 | 0.0007 |
|  | GO:0016477 | cell migration | 211 | 14 | 6.6 | 0.0005 |
|  | GO:0002686 | negative regulation of leukocyte migration | 5 | 2 | 40 | 0.0054 |
|  | GO:0043534 | blood vessel endothelial cell migration | 18 | 3 | 16.7 | 0.0085 |
| *Metabolism* | GO:0030799 | regulation of cyclic nucleotide metabolic process | 41 | 6 | 14.7 | 0.0004 |
|  | GO:0009190 | cyclic nucleotide biosynthetic process | 40 | 6 | 15 | 0.0003 |
|  | GO:0042445 | hormone metabolic process | 50 | 6 | 12 | 0.0012 |
|  | GO:0045937 | positive regulation of phosphate metabolic process | 51 | 6 | 11.8 | 0.0013 |
|  | GO:0034754 | cellular hormone metabolic process | 33 | 5 | 15.1 | 0.0010 |
|  | GO:0030814 | regulation of cAMP metabolic process | 37 | 6 | 16.2 | 0.0002 |
| *Transport and localization* | GO:0060341 | regulation of cellular localization | 127 | 10 | 7.8 | 0.0009 |
|  | GO:0015858 | nucleoside transport | 4 | 2 | 50 | 0.0033 |
|  | GO:0060627 | regulation of vesicle-mediated transport | 63 | 6 | 9.5 | 0.0038 |
|  | GO:0015833 | peptide transport | 41 | 5 | 12.2 | 0.0028 |
| *Hormone secretion* | GO:0046888 | negative regulation of hormone secretion | 13 | 3 | 23.1 | 0.0032 |
