# Supplementary material for: Global analysis of estrogen receptor beta binding to breast cancer cell genome reveals an extensive interplay with estrogen receptor alpha for target gene regulation
Source: BMC Genomics. 2011 Jan 14;12:36. doi: 10.1186/1471-2164-12-36 (PMC3025958; doi:10.1186/1471-2164-12-36)
Supplement: Additional File 6 — ERβ binding sites in proximity of miRNA loci. Containing the following information: miRNA name, ID of ER-β binding site upstream, Distance from the closest ERβ binding site upstream, ID of ER-β binding site downstream and Distance from the closest ERβ binding site downstream. [file 1471-2164-12-36-S6.DOC]

### **Additional Table 7. ERβ** binding sites in proximity of miRNA loci

| **miRNA name** | **ID ERβ binding site upstream** | **Distance from the closest ERβ binding site upstream*** | **ID ERβ binding site downstream** | **Distance from the closest ERβ binding site downstream*** |
| --- | --- | --- | --- | --- |
| hsa-mir-1204 | ERb_G8939 | -6961 | ERb_G8940 | 3904 |
| hsa-mir-1205 | ERb_G8955 | -7258 | - | - |
| hsa-mir-1207 | - | - | ERb_G8956 | 7727 |
| hsa-mir-1208 | ERb_G8958 | -7851 | ERb_G8959 | 2832 |
| hsa-mir-1244.chr2 | ERb_G5412 | -5319 | - | - |
| hsa-mir-1251 | - | - | ERb_G2227 | 8607 |
| hsa-mir-126 | - | - | ERb_G9461 | 9114 |
| hsa-mir-1285-2 | ERb_G4991 | -5608 | - | - |
| hsa-mir-1301 | ERb_G4831 | -5146 | - | - |
| hsa-mir-1302-5 | ERb_G5717 | -9762 | - | - |
| hsa-mir-135a-2 | - | - | ERb_G2228 | 4877 |
| hsa-mir-138-2 | - | - | ERb_G3390 | 2577 |
| hsa-mir-1469 | - | - | ERb_G3179 | 3634 |
| hsa-mir-149 | - | - | ER_G5470 | 1309 |
| cluster mir-183;96;182 | ERb_G8454 | -5444 from mir-183; -5689 from mir-96;  -9966 from mir-182 | - | - |
| hsa-mir-190 | - | - | ERb_G3032 | 8251 |
| hsa-mir-1910 | ERb_G3581 | -4620 | - | - |
| hsa-mir-193a | - | - | ERb_G3735 | 6445 |
| hsa-mir-203 | ERb_G2944 | -373 | - | - |
| hsa-mir-21 | ERb_G3918 | -3181 | ERb_G3919 | 1881 |
| hsa-mir-220a | ERb_G9637 | -7847 | - | - |
| hsa-mir-220c | ERb_G4667 | -8242 | ERb_G4666 | 5320 |
| cluster mir-23a;27a;24-2 | ERb_G4427 | -6998 from mir-23a; -7140 from mir-27a; -7298 from mir-24-2 | ERb_G4426 | 659 from 23a;  512 from 27a;  359 from 24-2 |
| hsa-mir-26b | ERb_G5367 | -3142 | - | - |
| hsa-mir-30a | ERb_G7839 | -42 | ERb_G7838 | 69 |
| hsa-mir-320b-1 | ERb_G477 | -3683 | ERb_G478 | 2492 |
| hsa-mir-320b-2 | ERb_G903 | -2868 | - | - |
| hsa-mir-326 | ERb_G1852 | -7501 | ERb_G1851 | 280 |
| hsa-mir-339 | - | - | ERb_G8059 | 1985 |
| hsa-mir-33b | - | - | ERb_G3699 | 1956 |
| cluster mir-34b;34c | ERb_G1953 | -27 from mir-34c | ERb_G1953 | 267 from mir-34b |
| hsa-mir-365-2 | ERb_G3737 | -1177 | ERb_G3738 | 5699 |
| hsa-mir-548c | - | - | ERb_G2178 | 2885 |
| hsa-mir-548d-2 | - | - | ERb_G4006 | 3532 |
| hsa-mir-548h-2 | ERb_G3283 | -6370 | - | - |
| hsa-mir-548p | ERb_G7346 | -6165 | - | - |
| hsa-mir-551a | - | - | ERb_G32 | 5175 |
| hsa-mir-554 | - | - | ERb_G557 | 2955 |
| hsa-mir-583 | - | - | ERb_G7338 | 6501 |
| hsa-mir-584 | - | - | ERb_G7476 | 7456 |
| hsa-mir-587 | ERb_G7887 | -1232 | - | - |
| hsa-mir-617 | ERb_G2209 | -5408 | - | - |
| hsa-mir-633 | - | - | ERb_G3968 | 5865 |
| hsa-mir-636 | ERb_G4109 | -412 | - | - |
| hsa-mir-637 | ERb_G4336 | -8002 | - | - |
| hsa-mir-641 | - | - | ERb_G4585 | 1994 |
| hsa-mir-642 | ERb_G4633 | -5670 | - | - |
| hsa-mir-648 | ERb_G6062 | -6964 | - | - |
| hsa-mir-649 | ERb_G6078 | -3590 | - | - |
| hsa-mir-661 | ERb_G9042 | -2952 | ERb_G9041 | 941 |
| hsa-mir-675 | - | - | ERb_G1540 | 6376 |
| cluster mir-941-1;941-2;941-3 | ERb_G5845 | -6294 from 941-1; -6601 from 941-2; -6713 from 941-3 | - | - |

* ERβ binding site within 10 kb from miRNA loci
